# Supplementary material for: Single-cell transcriptional profiling reveals cellular and molecular divergence in human maternal–fetal interface
Source: Sci Rep. 2022 Jun 28;12:10892. doi: 10.1038/s41598-022-14516-z (PMC9240006; doi:10.1038/s41598-022-14516-z)
Supplement: Supplementary file 1 — Supplementary Information 1. [file 41598_2022_14516_MOESM1_ESM.pdf]

Supplementary Fig 1. Information about the samples and the single-cell datasets quality.

a. Detailed information of human full-term placenta samples and single cell sequencing data.
b. The density graphic showing the distribution of detected gene number(left), unique feature counts (middle), and the percentage of mitochondrial counts (right)
c. Boxplot showing the expression pattern of canonical marker genes in each cell type.
d. Barplot showing the proportion of each sample in each cell cluster.
e. Table showing the sensitivity, accuracy, and specificity of discrimination function to infer the origin of fetal or maternal cells in full-term placenta.

Supplementary Fig 2. Molecular features analysis of STR with specific origin and spatial location.

a. Selected Gene Ontology (GO) terms identified by top 1000 highly expressed genes in each section of fetal origin (Left) and maternal origin (Right) STR cell. (Highly expressed genes with expressed cell number > 20%; gene coefficient of variability (CV) <1 and mean greater than 0 were used in each section).
b. Boxplot showing the differentially expressed genes of STR cells in each section. Two-sided Wilcoxon rank sum test were calculated, **** $P < 0.0001$.
c. Barplot showing the proportion of STR cells with determined origin and undetermined origin in each section.
d. Heatmap showing the expression pattern of genes related to cytokines and hormones in STR cells from different origin in each section.
e. Boxplot showing the different gene expression between STR cells from different origin in Mat_S. Two-sided Wilcoxon rank sum test were calculated, * $P < 0.05$, ** $P < 0.01$, *** $P < 0.001$, **** $P < 0.0001$.

Supplementary Fig 3. Integrated data analysis of trophoblast cell from our full-term placenta and downloaded first-trimester placenta.

a. T-distributed stochastic neighbor embedding (t-SNE) visualization of integrated data for full-term placenta single cell transcriptome data with that from published first-trimester placenta. On the right, barplot shows the proportion of full-term placenta cell and first-trimester placenta cell in each cluster.
b. Violin plot showing the expression of canonical marker genes for the defined cell types. Clusters annotated with the same cell type are shown together. (The clusters in Supplementary Fig 3a that each cell type includes are: CTB: 3, 16, 19, 21; EVT: 8, 11; STB: 29; STR: 7, 9, 13, 32; DEC: 4, 14, 18, 24, 27; PV: 6, 10, 12, 33; VEC: 20; LEC: 23; Dendritic cell, DC: 2, 5, 25; Hofbauer cell, HB: 17, 26, 28; T cell, TC: 1, 15; Natural killer cell, NK: 0, 22; Endometrial Epithelial Cell, EEC: 31)
c. t-SNE Plot showing the expression of canonical marker genes for the defined cell types of re-clustered trophoblast cells shown in Fig. 3a.
d. Location of each trophoblast subgroup of Fig. 3a on the trophoblast cell differentiation trajectory constructed by Monocle 2 (version 2.10.1).

Supplementary Fig 4. The features analysis in each EVT subgroup.

a. Boxplot showing the expression level of specific genes for each EVT subgroup.

b. Selected Gene Ontology (GO) terms identified by differentially expressed genes for each EVT subgroup.

Supplementary Fig 5. Cell type characterization of PE single cell transcriptome data and comparison of the differentially expressed key features between normal and PE specific cell types.

a. T-distributed stochastic neighbor embedding (t-SNE) visualization of single cell RNA transcriptome data of two selected preeclampsia(PE) placenta in reference 22, colors indicate different cell types or subtypes.
b. t-SNE plot showing the relative expression level of canonical marker genes for the defined cell types.
c. Barplot showing the proportion of each sample in each cellular subgroup.
d. Regulatory network of pregnancy-associated and candidate disease genes from Fig. 5a performed using STRING database.
e. Boxplot showing the relative expression levels of genes associated with trophoblast proliferation and differentiation in EVT subgroups between normal and PE sample.
f. Violin plot showing the relative expression levels for selected ligand-receptor pairs in EVT and VEC of normal and PE samples.
