# Supplementary material for: Single-cell transcriptional profiling reveals cellular and molecular divergence in human maternal–fetal interface
Source: Sci Rep. 2022 Jun 28;12:10892. doi: 10.1038/s41598-022-14516-z (PMC9240006; doi:10.1038/s41598-022-14516-z)
Supplement: Supplementary file 2 — Supplementary Figure 1. [file 41598_2022_14516_MOESM2_ESM.pdf]

Supplementary Fig.1

a

Sample information	Raw data information					Clean data information			
**Sample ID**	**Total cells**	**Mean Reads per Cell(K)**	**Number of Reads(M)**	**Median Genes per Cell**	**Fiftered cells**	**Remained total cells**	**Total cells**	**Rate of passed cells (%)**	**Sequencing saturation (%)**
FS_a	797	1435	1100	1753	111	686	3138	86.1	97.0
Mid_S_a	3260	585	1900	1686	808	2452		75.2	97.1
FS_b	1740	381	660	2019	109	1631	4030	93.7	95.7
Mid_S_b	936	1730	1600	1666	288	648		69.2	98.2
Mat_S_b	1889	643	1200	2554	138	1751		92.7	94.8
FS_c	1391	829	1200	2817	218	1173	4270	84.3	93.7
Mid_S_c	2709	378	1020	3561	505	2204		81.4	88.0
Mat_S_c	1025	966	990	2616	132	893		87.1	92.7
**total**	**13747**					**11438**		**83.2**	**94.7**

FS: Fetal section, Mid_S: Middle section, Mat_S: Maternal section; a,b,c representing three individuals respectively

b

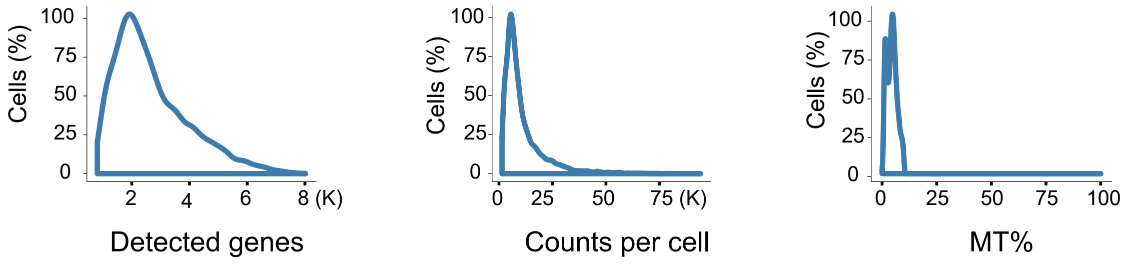

c

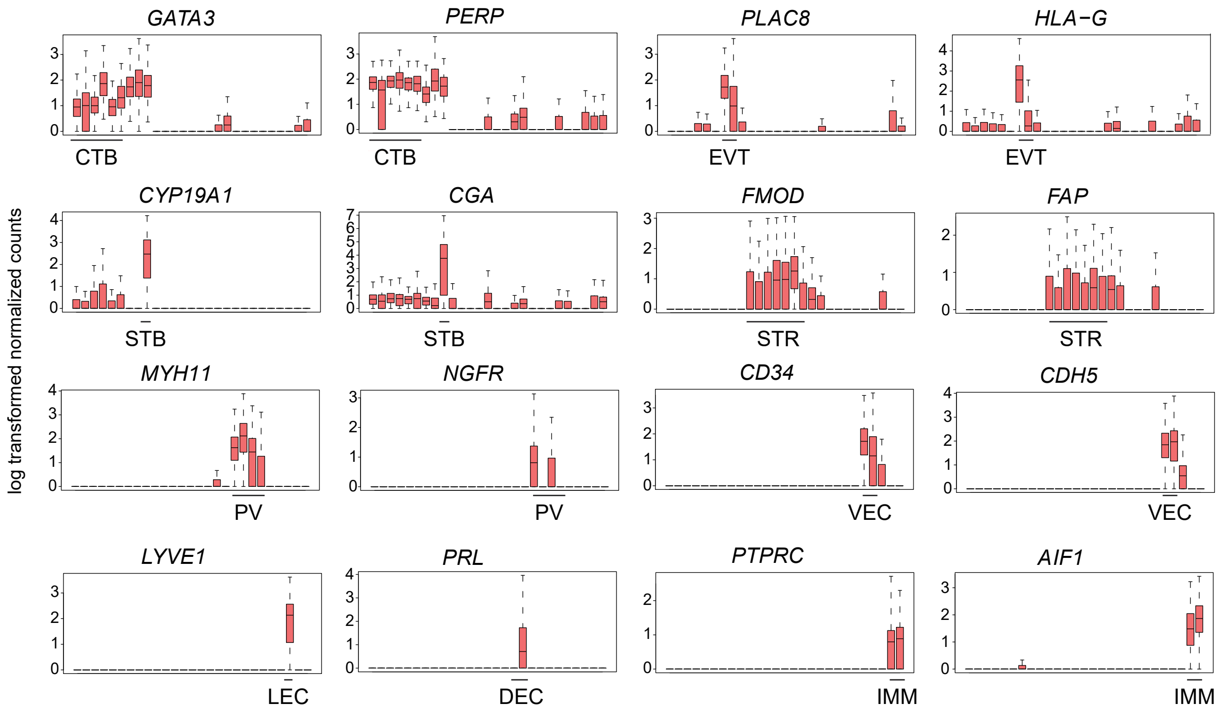

d

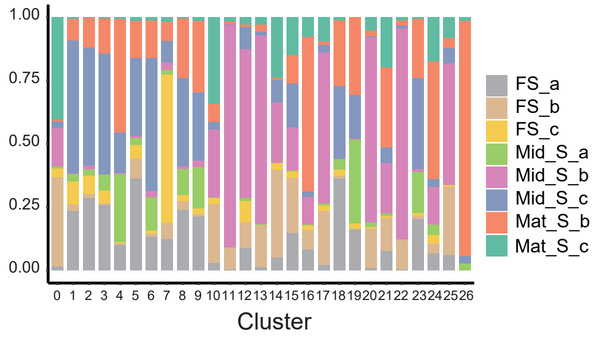

e

Section	Orign	Sensitivity (%)	Specificity (%)	Accuracy (%)
FS	Fetal	90.8	92.1	91.1
	Maternal	96.8	89.5	91.3
Mid_S	Fetal	92.0	86.6	91.3
	Maternal	91.9	91.1	95.7
Mat_S	Fetal	95.2	93.7	94.5
	Maternal	99.2	93.5	91.2
