# Supplementary figures and images for: Single-cell transcriptional profiling reveals cellular and molecular divergence in human maternal–fetal interface
Source: Sci Rep. 2022 Jun 28;12:10892. doi: 10.1038/s41598-022-14516-z (PMC9240006; doi:10.1038/s41598-022-14516-z)

Supplementary Fig.2

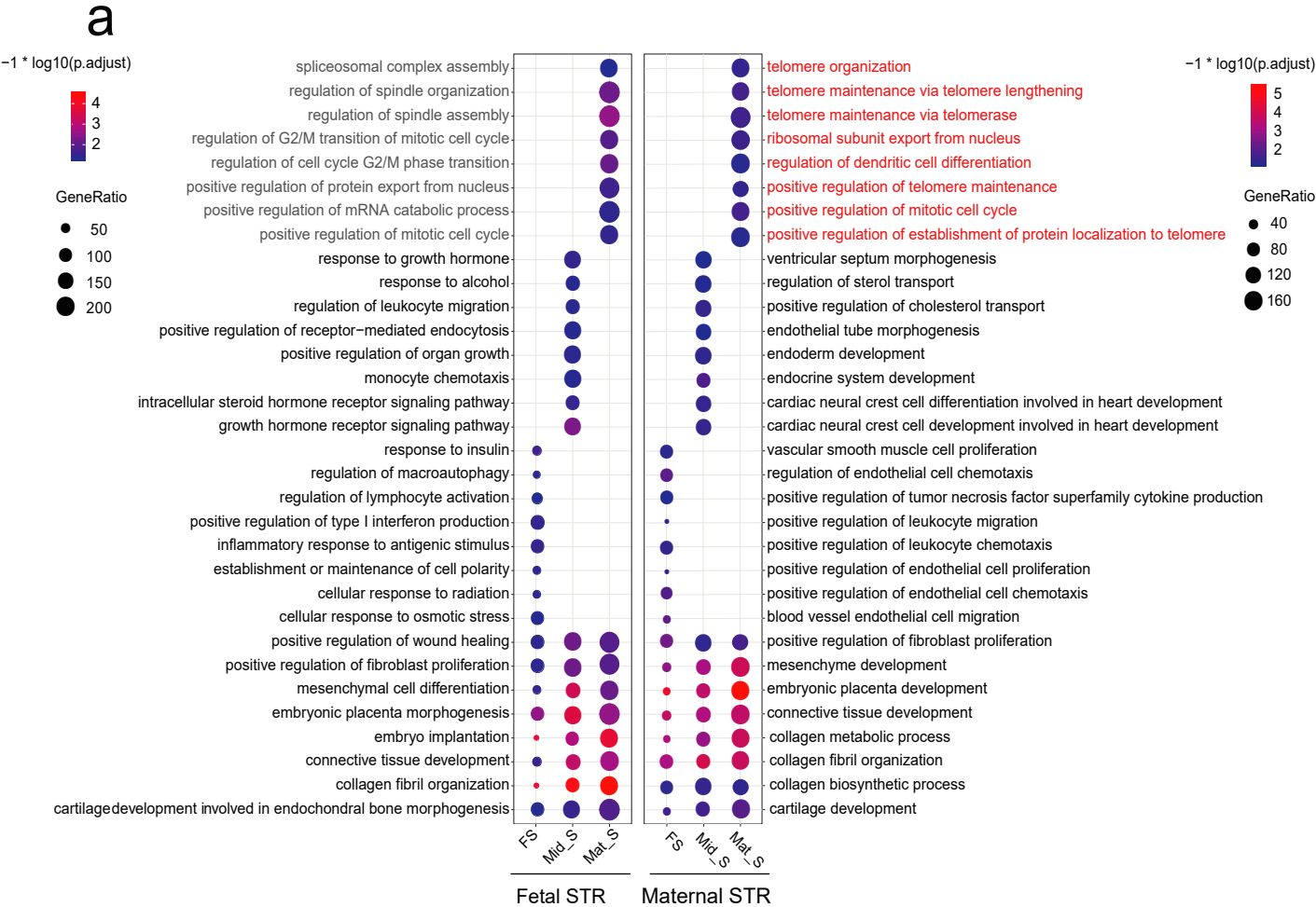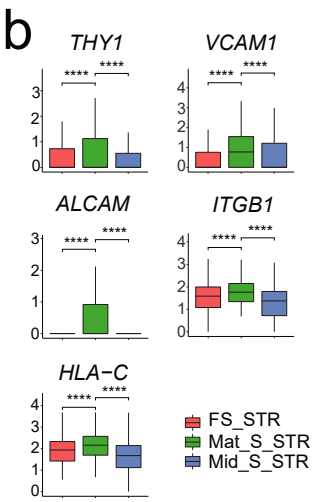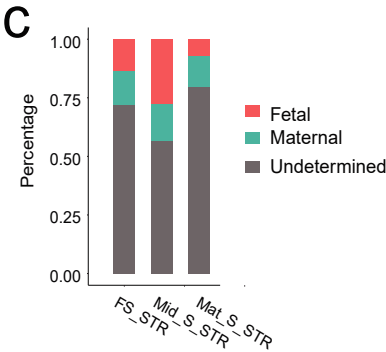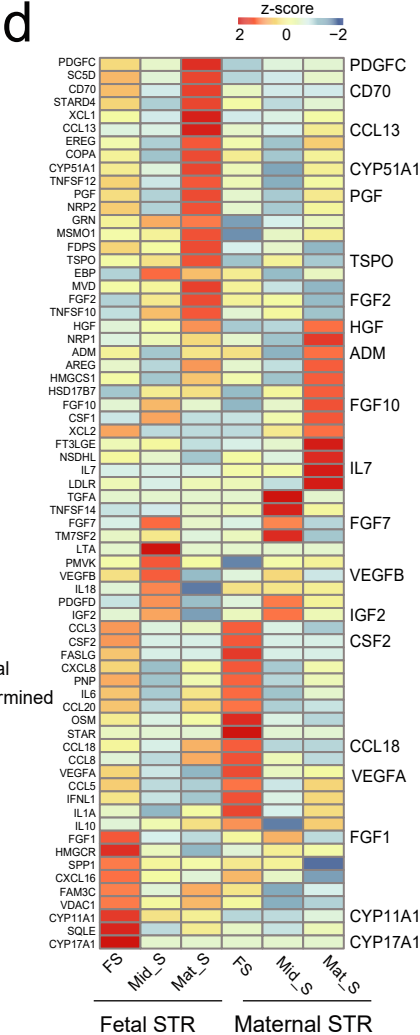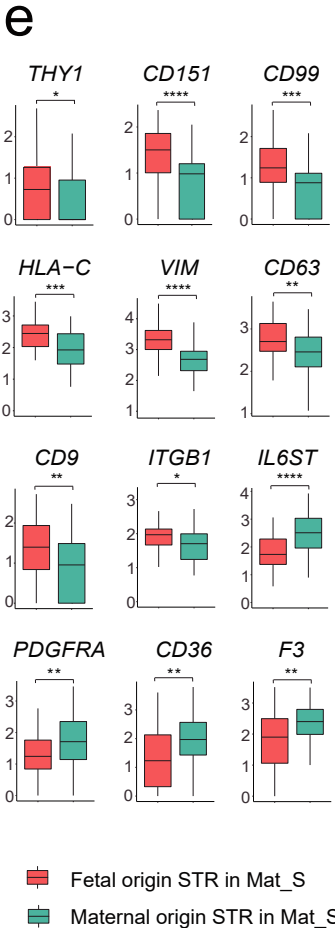

Supplement: Supplementary file 3 — Supplementary Figure 2. [file 41598_2022_14516_MOESM3_ESM.pdf]

Supplementary Fig.3

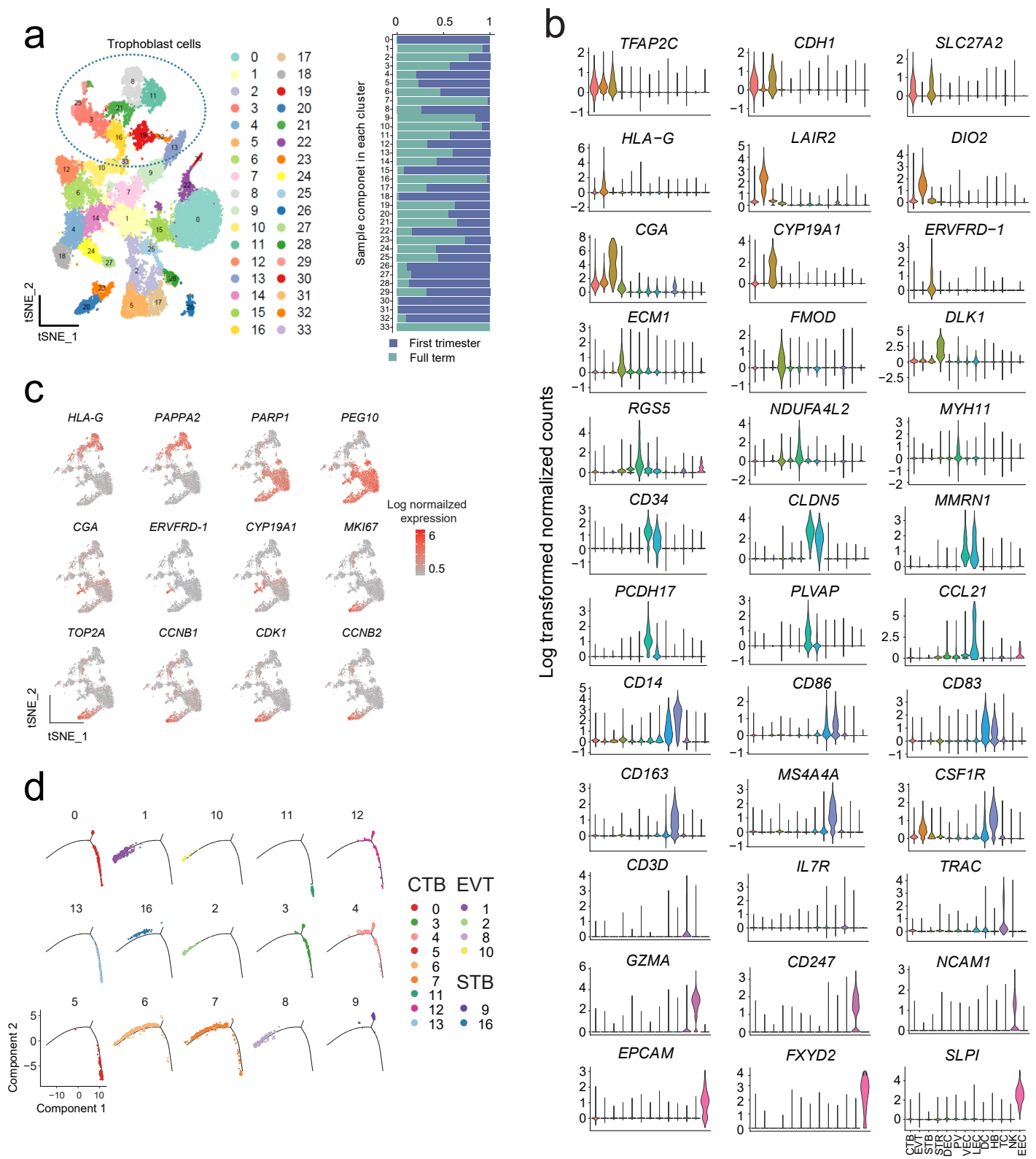

Supplement: Supplementary file 4 — Supplementary Figure 3. [file 41598_2022_14516_MOESM4_ESM.pdf]

# Supplementary Fig.4

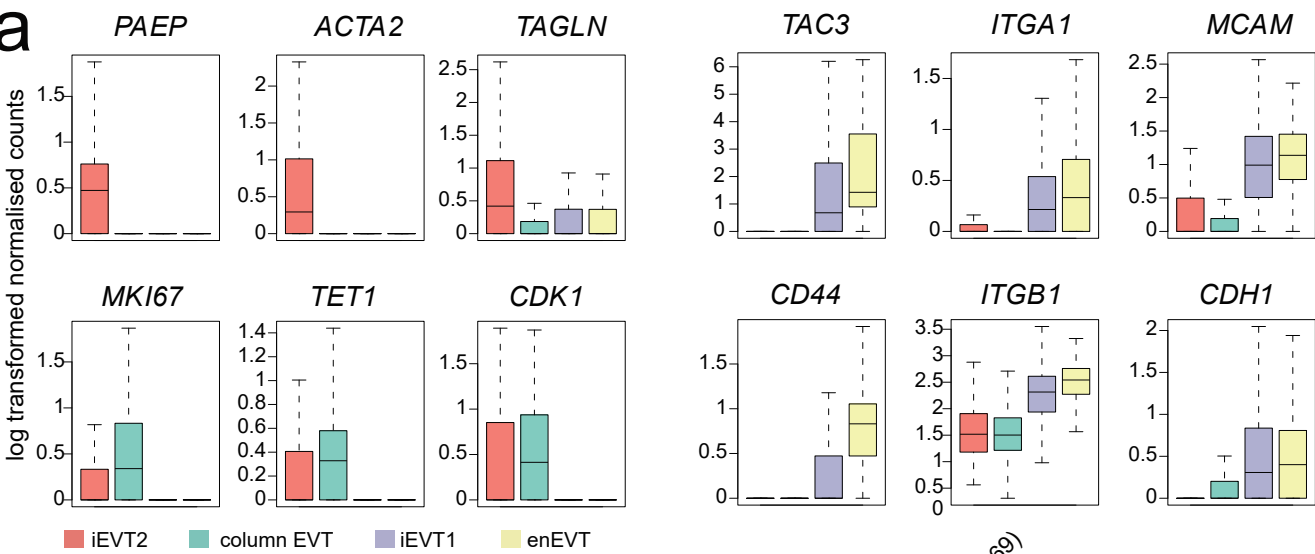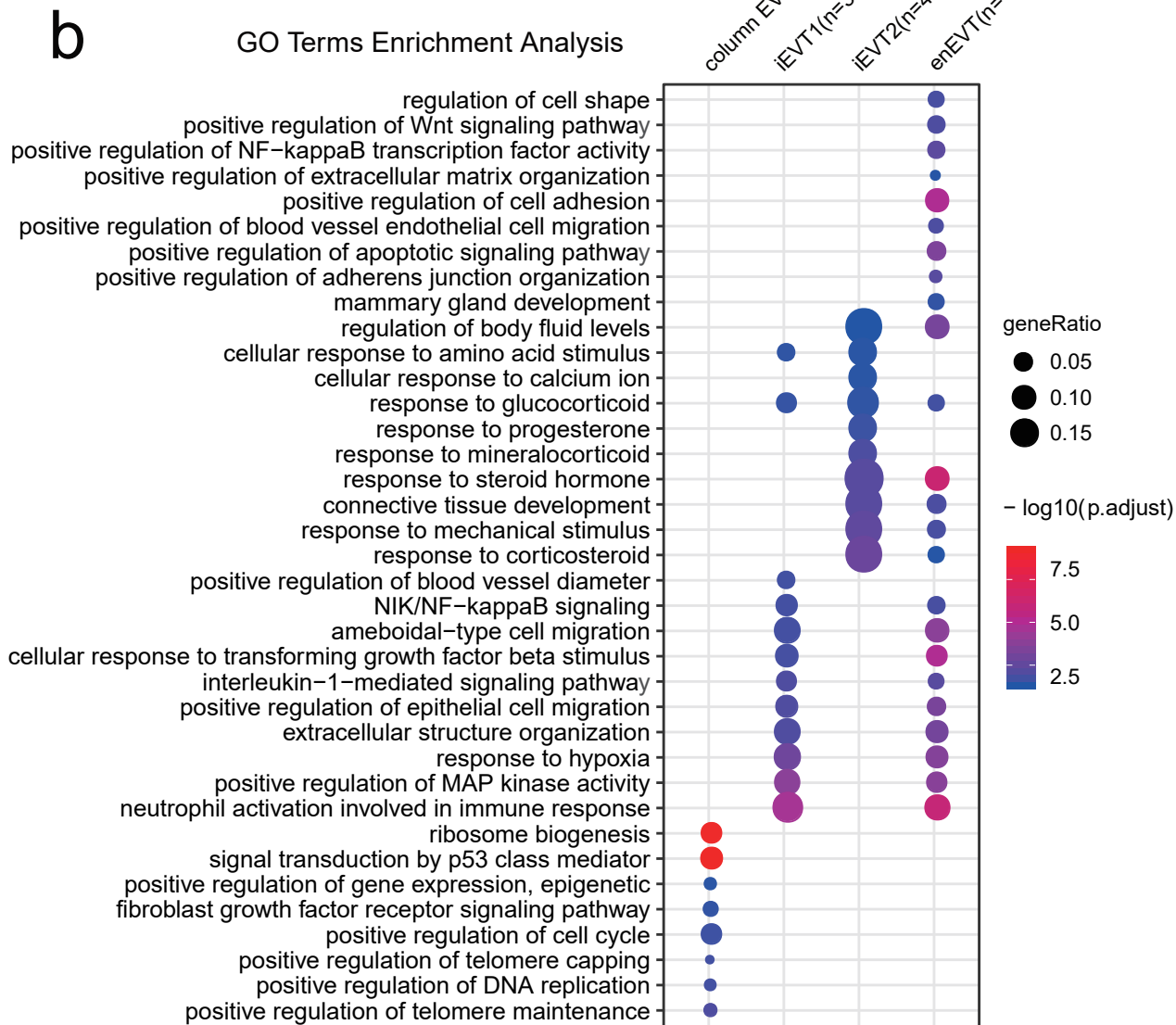

Supplement: Supplementary file 5 — Supplementary Figure 4. [file 41598_2022_14516_MOESM5_ESM.pdf]

Supplementary Fig.5

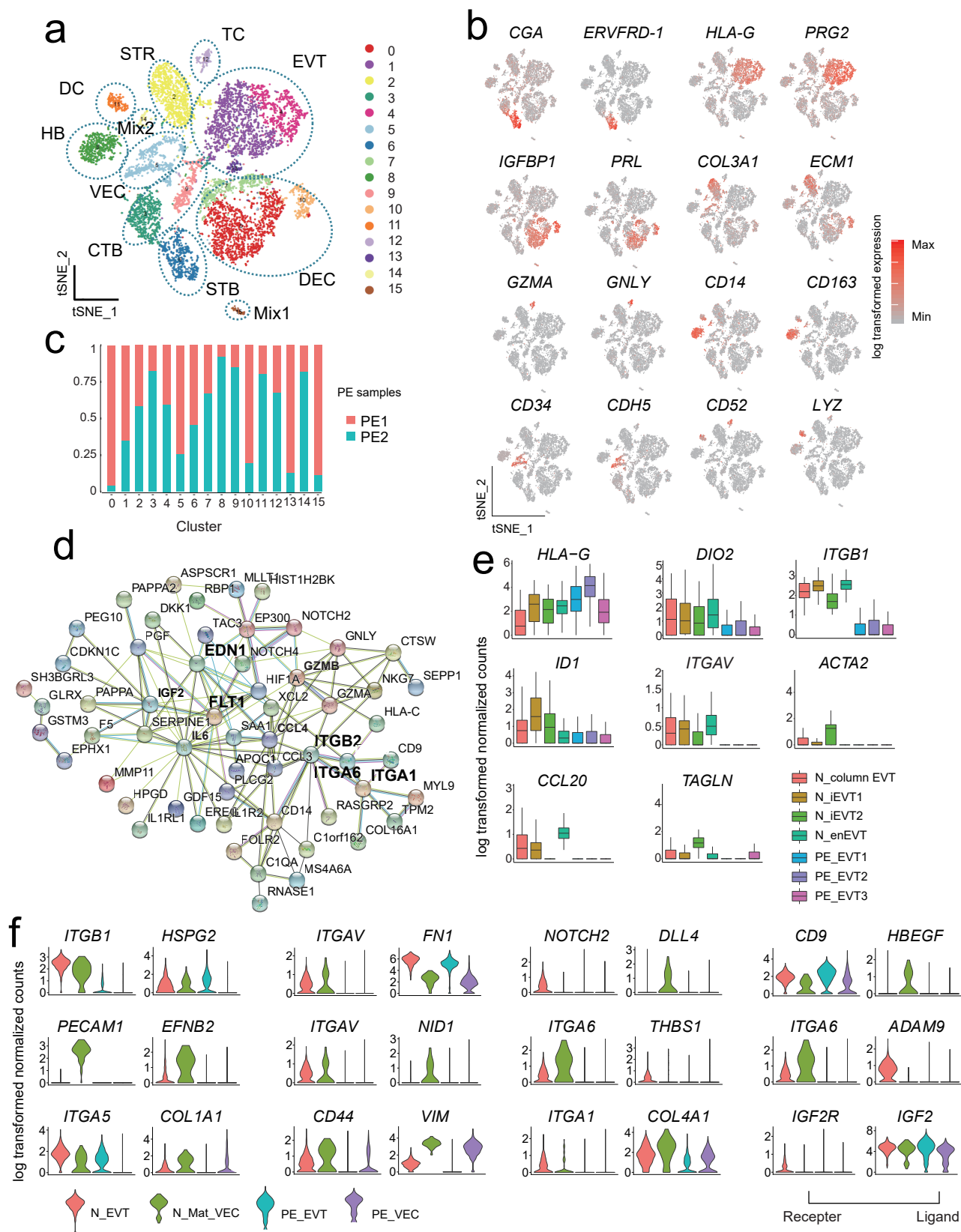

Supplement: Supplementary file 6 — Supplementary Figure 5. [file 41598_2022_14516_MOESM6_ESM.pdf]
